# How to perform a high-quality peer review

**DOI:** 10.46989/001c.128601

**Published:** 2025-02-03

**Authors:** Mohamad Mohty, Junia V. Melo

**Affiliations:** 1 Sorbonne Université; Centre de Recherche Saint-Antoine, INSERM UMRs 938; Service d’Hématologie Clinique et de Thérapie Cellulaire, Hôpital Saint Antoine, AP-HP, Paris, France; 2 Faculty of Health and Medical Sciences, University of Adelaide, SA 5000, Australia

**Keywords:** peer-review, reviewer, integrity, medical literature, scientific research

## Abstract

High-quality peer review is a cornerstone of credible and impactful scientific and medical publishing. This manuscript provides a comprehensive overview of the best practices, responsibilities, and evaluation criteria for peer reviewers in clinical and translational research. By adhering to high standards of objectivity, rigor and professionalism, peer reviewers support the integrity of scientific research and contribute to the evolution of evidence-based medicine. We elaborate on the principles and structured processes that ensure a thorough, impartial review, aiming to guide reviewers in producing evaluations that enrich the scientific discourse and foster innovation in clinical practice.

## Introduction

The peer review process is a critical component of scientific research and publishing, designed to validate and improve manuscripts before their public release. Peer review serves as a quality control mechanism, ensuring that only robust, accurate and meaningful scientific work is published. For medical journals, where publications can influence patient care and inform policy decisions, the integrity of the peer review process is particularly vital. By refining data interpretation and enhancing the clarity of research findings, peer review upholds the standards of clinical and translational research, benefiting both the scientific community and society at large.

## Core principles of high-quality peer review

The effectiveness of peer review rests on at least three foundational principles which include (i) disclosure of conflicts of interest: to maintain transparency, reviewers must disclose any potential conflicts of interest, including financial, professional, or personal biases that could affect their impartiality. Disclosing these conflicts protects the integrity of the peer review process and maintains trust among authors, editors, and readers; (ii) scientific expertise: reviewers must have sufficient knowledge in the manuscript’s subject area to provide a useful assessment of its content. This expertise ensures that reviewers can evaluate the scientific rigor, relevance, and quality of the research appropriately; and (iii) constructive feedback: feedback should not only highlight strengths and weaknesses, but also provide actionable recommendations for improvement. Constructive feedback allows authors to refine their work, and often strengthens the study’s contribution to its field (Table 1).

**Table 1.** Summary of the principles, responsibilities and best practices of peer review

**Principles of high-quality peer review**	**Reviewer’s responsibilities**	**Best practices for reviewers**
Disclosure of conflicts of interest	Validation of data and conclusions	Professionalism and respectfulness
Scientific expertise	Evaluation of data integrity, novelty and impact, and methodological soundness	Clear and constructive tone
Constructive feedback	Identification of improvement areas and constructive feedback	Timeliness
	Impartial evaluations based on the scientific content exclusively	Suggestions for improvement should be feasible
	Structured review	

## Reviewer’s responsibilities

The reviewing of a manuscript must be thorough and methodical. It must encompass the assessment of the entire manuscript, including careful scrutiny of the tables, figures and any supplementary data. This requires dedicated time and attention to detail, the absence of which results in a poor, misleading and even ‘useless’ review. Peer reviewers play a crucial role in ensuring the credibility and scientific value of published research. Their primary responsibilities include validation of data and conclusions where reviewers must meticulously examine the data and confirm that the conclusions drawn are well-supported. They should be able to spot incomplete or inadequate results and, in particular, overinterpretation of such in the Discussion section of a report. This validation process is essential for maintaining the scientific accuracy of published findings. Thus, effective peer review involves systematic evaluation of the manuscript data integrity, novelty and impact, and methodological soundness. Reviewers should confirm that the data are presented consistently and accurately. They should identify any gaps or inconsistencies in data reporting that could undermine the study’s findings. Evaluating the manuscript’s novelty involves assessing the uniqueness of its findings and the value they add to the existing body of knowledge. Impact refers to the study’s potential influence on clinical practice, policy, or future research. Finally, it is up to the reviewers to ensure that the study’s methodology is appropriate and statistically sound. This includes evaluating whether the study design is suitable for the research question, the adequacy of sample sizes, and the correctness of statistical analyses.

Identification of improvement areas is another key issue. As part of the constructive feedback, it is the reviewer’s responsibility to provide specific suggestions for improvement which can contribute to the manuscript’s clarity, methodological robustness, and overall impact. The assessment of relevance is another essential task of the peer review process, encompassing the evaluation of whether the research is novel and significant enough to warrant publication. These are the most important aspects of a paper and underpin the reason for it to be published. The reviewer must systematically have this in mind for any and all papers he/she is asked to assess. Therefore, consideration of both the originality of the findings and their potential to advance the field should be paramount in the reviewing process.

Finally, reviewers must offer impartial evaluations based on the scientific content rather than on factors such as the author’s institution or status. Deviation from the required impartiality constitutes a conflict of interest and is unacceptable. An unbiased review is critical to preserving the integrity of the scientific publishing process (Table 1).

## Structured review process

A well-organized, systematic approach to reviewing manuscripts ensures that each section is thoroughly and consistently evaluated. The review process typically involves a section-by-section analysis:

- Title and abstract: reviewers should ensure that the title is accurate and reflective of the study’s content. In the interest of conciseness and expressiveness, the reviewer can suggest alternative wording and/or focus for the title, and should strongly discourage long, discursive titles. The abstract should be checked for its essential role of providing a concise, standalone summary of the research question, methods, results, and conclusions.
- Introduction: this section should establish the study’s background and rationale. Reviewers should assess whether the authors have cited relevant literature and clearly outlined the study’s purpose.
- Methods: in this section, reviewers should verify the ethical soundness of the study design, the appropriateness of the methodology, and the adequacy of the statistical approach. It is important to note that a major role of the Methods section on a paper is to ensure the work/analysis can be reproduced and validated at independent research/clinical centers. Therefore, the reviewer should pay particular attention as to whether detailed, complete and retrievable information for testing such reproducibility are given on the manuscript (or its supplementary data appendix).
- Results: reviewers must ensure that data are presented transparently and unambiguously, and that the findings are consistent with the study objectives. Repetition of the same information on both the free-flow text and tables should be strongly discouraged. This type of unnecessary and distracting redundancy should be specifically scrutinized and criticized by the reviewers on their comments and suggestions to the authors. Thus, if a table includes extensive details on each set of results, the text under Results should only provide the summarized description of those data, with reference to the table for the additional details.
- Discussion: reviewers should evaluate the accuracy of the interpretation, including whether the authors acknowledge study limitations and consider alternative explanations for their findings. They should also check that the conclusions are well-founded and proportionate to the data presented.
- Figures and tables: effective visual elements such as figures and tables should stand alone, meaning they should be understandable without needing to refer back to the text. This also applies to the need for definition of abbreviations for each table and figure, regardless of whether they were already used and defined on the manuscript body. Reviewers should verify that these elements are accurate, non-redundant, and aligned with the content.
- General features and requisites: each journal has its own set of rules and detailed instructions to authors. These dictate adherence to standardized writing and graphics features which are essential for a manuscript’s capacity to convey the message in an understandable, uncontroversial and clearly visible way. Examples of such requirements are the necessity to define all abbreviations at their first mention, and the attention to the preparation of tables and figures using font sizes of sufficient legibility. Reviewers must scrutinize both features and raise criticisms if they are not compliant with the journal rules.

## Best practices for reviewers

Professionalism and respectfulness are essential traits in a reviewer. High-quality reviews require that reviewers maintain a clear and constructive tone. Based on the outlined criteria, a reviewer should be equipped to recommend minor revisions, major revisions, or rejections. These recommendations should be guided solely by the quality and rigor of the manuscript without being influenced by the journal’s impact factor or the authors’ reputation. Additional best practices include timeliness, which implies completing reviews within 10-14 days to help facilitate prompt editorial decisions. The latter is essential in fields where the rapid dissemination of knowledge is critical. Also, the suggestions for improvement should be feasible. Reviewers should provide practical suggestions that are realistic and aimed at enhancing the manuscript’s presentation, methodological rigor, or interpretative clarity. This includes offering advice on data visualization, argument clarity, and referencing accuracy (Table 1). If a specific improvement cannot be incorporated into the study, it should be explicitly acknowledged in the limitations. Thoroughly discussing these limitations enhances transparency, clarifies the study’s scope and findings, and provides a clearer context for interpreting the results.

## Benefits and challenges of peer review

The peer review process benefits both the individual reviewer and the broader scientific community. From an individual point of view, reviewers gain early access to cutting-edge research, which can contribute to their educational growth and professional development. Participating in peer review also provides insight into the editorial process, enhancing the reviewers’ skills in critical evaluation and scientific writing. From a societal perspective, peer review ensures that high-quality, evidence-based findings are published, promoting the integrity and credibility of scientific literature. This process is crucial for fields like medicine, where published research can influence clinical decisions and impact patient care.

Despite its importance, the peer review process faces several challenges that must be addressed to maintain and enhance its effectiveness:

- Recognition and incentives for reviewers: although peer review is a time-intensive task, it often goes unrecognized. There is growing interest in providing tangible rewards for high-quality reviews, such as acknowledgment in journals or credits for continuing education. These incentives could encourage greater participation and higher-quality reviews.
- Bias and integrity: ensuring unbiased evaluations is a challenge, particularly when reviewers may be unconsciously influenced by factors such as the authors’ institution or their established ‘pedigree’ in the clinical and academic world. Strategies to mitigate bias, including training and completely blinded review processes, can help address these issues and maintain the review’s integrity.
- ‘Official’ training: scientific/medical writing is an absolutely essential part of a clinician’s and/or academic health professional’s life. Yet, it is currently a neglected area in the formal education of such professionals. The same deficit occurs in the preparation of individuals for the acquisition of peer-review skills. It is therefore clear that such an omission in our university education system should be urgently addressed, and well-designed modules encompassing both skills should be implemented in medical and science curricula.

## Conclusion

High-quality peer review is fundamental to the advancement of scientific knowledge. By adhering to rigorous standards, providing constructive feedback and maintaining professionalism, reviewers contribute to a process that safeguards the credibility of research. This manuscript highlights the principles, responsibilities, and best practices that are essential to the classical peer review process. However, the current manuscript does not address emerging models of peer review, such as preprint publishing with open commenting, nor does it explore how these approaches could influence the future of the peer-review process. As the scientific landscape continues to evolve, fostering a culture of excellence in peer review remains a priority. The integrity of clinical and translational research relies on a commitment to thorough, unbiased, and respectful peer evaluation, ensuring that published research meets the highest standards and positively impacts science and society.

### AUTHORSHIP

MM wrote the first draft of the manuscript, and both authors commented on and edited subsequent versions. Both authors read and approved the final manuscript.

### STATEMENTS AND DECLARATIONS

The authors declare no competing financial interests concerning this work.

### ETHICAL APPROVAL

Not applicable.

### CONSENT TO PARTICIPATE/INFORMED CONSENT

Not applicable.

### CONSENT FOR PUBLICATION

Not applicable.

